# Exploring the therapeutic potential of bimodal stimulation for tinnitus

**DOI:** 10.1097/MS9.0000000000002998

**Published:** 2025-02-06

**Authors:** Ali Aamir Khan, Hamid Nasim, Gaffar Alemam A. Manhal, Khabab Abbasher Hussien Mohamed Ahmed

**Affiliations:** a Department of Otorhinolaryngology, Ayub Medical College, Abbottabad, Khyber Pakhtunkhwa, Pakistan; b Faculty of Medicine, University of Khartoum, Khartoum, Sudan

**Keywords:** bimodal neuromodulation therapy, bimodal stimulation, ENT, therapeutic potential, tinnitus

## Abstract

**Background:**

Tinnitus is the perception of sound without any external acoustic stimuli, affecting over 10% of the population, especially older adults. Current treatments focus on symptom management rather than a complete cure, often providing limited relief.

**Objective:**

To evaluate the efficacy and safety of bimodal neuromodulation therapy for tinnitus, compared to traditional treatment methods.

**Results:**

Bimodal neuromodulation, which combines sound and tongue stimulation, has shown significant improvement in tinnitus symptoms. FDA-approved studies report sustained symptom reduction in over 77% of patients for up to 12 months. The most common side effect is an increase in tinnitus symptoms, similar to sound-only treatments.

**Conclusion:**

This therapy presents a high benefit-to-risk ratio with minimal side effects, offering better clinical outcomes and patient satisfaction compared to existing treatments. Adjustments in treatment parameters can further enhance its effectiveness over time. Bimodal neuromodulation shows promise as a more effective treatment for tinnitus. Future clinical trials should replicate these positive findings, evaluate long-term effects, and compare their effectiveness to other treatment options to improve accessibility and clinical outcomes for tinnitus patients.

## Introduction

Tinnitus, the perception of sound in the absence of external stimuli, is a complex and multifaceted condition that significantly affects the quality of life of millions worldwide. The term tinnitus originates from the Latin word tinnire, meaning “to ring,” and describes auditory perceptions such as ringing, buzzing, and hissing that are often distressing and challenging to manage^[^1^]^. People suffering from tinnitus may experience auditory perceptions that cannot be explained and can include but are not limited to sounds like hissing, buzzing, pulsations, ringing, sizzling, and clicking. It may result from acoustic trauma, chronic impairment of hearing, and mental stresses, as well as manifest spontaneously^[^2^]^. Unlike auditory hallucinations, the sounds perceived in tinnitus are vague and devoid of meaning. Tinnitus can be subjective when the experience is unique to the person or objective when an observer can hear the tinnitus^[^3^]^. Tinnitus can be classified as either primary or secondary depending on the underlying cause and can be acute or chronic depending on the duration of symptoms^[^4^]^.

## Prevalence and impact

Tinnitus affects more than one in ten people in the general population, with the highest prevalence among older adults. The prevalence and incidence of tinnitus varied by sex, age, continent, and definition of tinnitus^[^1-5^]^. It has been shown that tinnitus affects more than 740 million adults in the world and it is perceived as a major problem by more than 120 million people (mostly elderly ones) aged 65 years or older^[^5^]^. A study also suggested that tolerable and annoying tinnitus were experienced by 15.4% and 12.2% of participants, respectively. Additionally, the severity of tinnitus has been associated with comorbid conditions such as hypertension, type 2 diabetes, and cardiovascular disease, with a notable link to poor mental health and quality of life. Annoying tinnitus was significantly associated with depressive mood, psychological distress, suicidal ideation, and impaired health-related quality of life in all dimensions after controlling for confounding variables. Participants with annoying tinnitus had significantly lower EQ-5D index scores than those without tinnitus. These findings suggest that annoying tinnitus may harm older adults’ mental health and quality of life^[^6^]^.

## Diagnosis of tinnitus

Most tinnitus is subjective and experienced only by the patient; consequently, the diagnosis of tinnitus primarily depends on patient self-reports. Objective tinnitus is infrequent and audible to the examiner, making diagnosing it easier. In the vast majority of cases, objective tinnitus is associated with vascular abnormality; therefore, its diagnosis is frequently founded on radioactive imaging examinations such as magnetic resonance imaging (MRI) or computed tomography (CT)^[^7^]^. Only certain situations, such as unilateral or pulsatile tinnitus, asymmetrical loss of hearing, a focal neural deficit, otosclerosis, or head injuries, warrant imaging of the head and neck in the patient. The advised diagnostic exams include an MRI of the cerebellopontine angle and internal acoustic meatus for asymmetrical hearing loss, CT of the temporal bone for possible cholesteatoma or otosclerosis, and CT of the tinnitus for pulsatile tinnitus.^[^4^]^

## Treatment of tinnitus

Currently, no drug or treatment is clinically effective enough for this condition. Common interventions include counseling, cognitive behavioral therapy, tinnitus retraining therapy, and sound therapy, all of which aim to alleviate distress and improve quality of life^[^1^]^. This article, therefore, aims to provide a brief overview of the current evidence and challenges regarding the efficacy and safety of bimodal stimulation for tinnitus patients highlight the gaps and limitations in the existing literature and suggest directions for future research on this topic.

Current treatment modalities of tinnitus aim at either masking tinnitus or complete cessation. Tinnitus masking therapies include hearing aids, sound, cognitive behavioural, tinnitus retraining, and music therapies. Tinnitus cessation therapies include surgical treatments such as cochlear implants, microvascular decompression, and noninvasive neuromodulation, but overall, none of these therapies aimed at cessation of tinnitus are guaranteed to be effective, and treatment outcomes remain fairly controversial^[^8^]^.

## Bimodal neuromodulation therapies

Bimodal neuromodulation devices are an emerging and promising solution for tinnitus^[^8-11^]^. The most common approach combines sound stimulation with electrical or mechanical stimulation to induce neural plasticity and reduce tinnitus perception: (i) ***Sound Stimulation***: Bimodal neuromodulation devices use external sound stimuli to provide auditory stimulation. They typically incorporate a pair of earphones or speakers to deliver specific sound patterns or tones to the individual experiencing tinnitus. These sounds can be customized based on the individual’s tinnitus frequency or characteristics. The purpose of sound stimulation is to partially mask or distract an individual from the tinnitus perception. In other words, sound stimulation enables external noise to mask tinnitus, hence, distracting patients and decreasing the contrast between the tinnitus signal and the background activity of the auditory system^[^9^]^. (ii) ***Electric Stimulation***: Electric nerve stimulation modulates neural pathways involved in tinnitus perception by leveraging neuroplasticity, the brain’s ability to reorganize itself through new neural connections. Tinnitus is often associated with hyperactivity in the auditory system, particularly in the dorsal cochlear nucleus and auditory cortex. Electric stimulation, such as transcutaneous electrical nerve stimulation (TENS) or transcranial direct current stimulation (tDCS), reduces this hyperactivity, promoting a balance between excitation and inhibition through the activation of inhibitory interneurons^[^1,11^]^. This process facilitates long-term potentiation and long-term depression, altering synaptic strengths in auditory pathways to reduce the perception and distress of tinnitus. Additionally, electric stimulation targets interactions between the auditory and somatosensory systems, particularly in the dorsal cochlear nucleus, and can suppress tinnitus symptoms when paired with somatosensory inputs, as seen in bimodal neuromodulation^[^11^]^. Studies, such as randomized trials by Conlon *et al* (2020, 2022)^[^11,12^]^ (*n* = 326, *n* = 191) have demonstrated sustained improvements in tinnitus severity for over 12 months, highlighting the therapeutic potential of electric nerve stimulation. Electric nerve stimulation for tinnitus therapy involves specific parameters, including stimulation duration, frequency, and intensity. Clinical trials have typically used sessions lasting between 30 to 60 minutes daily over a span of 12 weeks, yielding effective symptom relief^[^11,12^]^. The frequency of electrical stimulation varies, with tDCS generally applying low-intensity currents of 1–2 mA, and TENS utilizing frequencies between 10 and 100 Hz^[^1,4^]^. Additionally, combining sound stimulation personalized to the tinnitus frequency with synchronized tongue stimulation has demonstrated substantial benefits. Studies have shown that fine-tuning these parameters, such as adjusting stimulation settings after 6 weeks to address habituation, significantly enhances outcomes^[^11,12^]^.

Other approaches have been explored, including skin vibration therapy. Skin vibration therapy, which delivers mechanical vibrations to the skin (typically in the neck or head area), has been shown to engage the somatosensory system and may influence auditory processing pathways. Studies suggest that this form of stimulation, when combined with sound therapy, can help modulate tinnitus perception by providing additional sensory input that distracts from or masks tinnitus sounds^[^13,14^]^.

These multimodal approaches have shown promise in clinical trials, with some studies demonstrating significant improvements in tinnitus symptoms, particularly when the therapies are tailored to individual needs^[^12^]^.

Bimodal neuromodulation devices aim to achieve therapeutic effects such as auditory masking, promoting neural plasticity, and facilitating habituation to the tinnitus perception. Bimodal neuromodulation devices are particularly helpful for patients with tinnitus, but they can also be useful in the treatment of other conditions, such as chronic pain and depression^[^8,9^]^. In light of recent studies, the FDA has granted de novo approval to a bimodal neuromodulation device for tinnitus treatment.

## Evidence and practical considerations

Bimodal neuromodulation combines sound and tongue stimulation, showing significant improvements in tinnitus symptoms compared to sound therapy alone^[^9,11^]^. The study looked at the benefits of bimodal neuromodulation, a treatment for tinnitus (ringing in the ears). They combined the data from three group participants who received different types of Therapy^[^11^]^. They found that most participants (over 81%) experienced an improvement in their tinnitus symptoms after 12 weeks of treatment, and this improvement was sustained for over 12 months (over 77% of participants). However, around 20% of participants did not experience any progress or even had an increase in their symptoms. They measured the improvement using two scales, Tinnitus Handicap Inventory (THI) and Tinnitus Functional Index (TFI) and found that, on average, there was a decrease of around 14 points in THI and 13.6 points in TFI at the end of treatment^[^4^]^. This improvement was sustained for over 12 months. These results are based on a large sample size of over 151 participants^[^4^]^. In the TENT-A1 study, the use of a bimodal neuromodulation approach showed a significant reduction in tinnitus symptoms in the first 6 weeks, with 80% of patients reporting significant improvements. In the following 6 weeks, the results showed fewer improvements due to habituation. In the TENT-A2 study, it has been found that if we adjust the bimodal stimulation parameter settings to overcome the habituation effects in the second 6 weeks of treatment, it further alleviates the symptoms significantly with its therapeutic effects staying for the span of 12 months^[^12^]^. The bimodal neuromodulation approach to tinnitus has shown a high benefit-to-risk ratio with no severe treatment-related side effects. The most common side effect reported was an increase in tinnitus symptoms. Still, it is not notably different from the adverse impacts of sound-only treatment, suggesting that it is related to the sound component of the therapy without any link to tongue stimulation^[^8,9^]^. With their high satisfaction and compliance rates, bimodal neuromodulation devices can bring dramatic changes to the clinical outcomes of tinnitus as compared to current treatment choices without compromising the safety of patients because of its minimal side effects and long-term results. Although bimodal neuromodulation devices have shown promising results for bimodal stimulation in reducing tinnitus symptoms, more research is needed to determine the efficacy of devices, optimal stimulation parameters and the long-term effects of this therapy.

## Future directions

Future studies should also investigate the potential of bimodal stimulation for treating other conditions. Future recommendations for the utilization of bimodal stimulation in tinnitus management should focus on the development of personalized treatment plans that account for the heterogeneous nature of tinnitus among individuals. It is essential to advance bimodal stimulation devices to improve their design and functionality, allowing for more precise and targeted stimulation. By tailoring treatment strategies and optimizing device technology, the efficacy of bimodal stimulation therapy in tinnitus management can be enhanced, leading to improved patient outcomes and a better understanding of its therapeutic potential. It is necessary to investigate the neural mechanisms of bimodal stimulation to gain a better understanding of how it works and how it can be optimized for maximum therapeutic benefit. Overall, bimodal stimulation shows promising potential as a treatment for tinnitus, and further research is needed to optimize its therapeutic potential and develop personalized treatment plans.

## Conclusion

Tinnitus is a pervasive condition with limited effective treatments, but the advent of bimodal neuromodulation therapies offers hope for better management. These therapies, which combine sound and electrical or mechanical stimulation, have demonstrated efficacy in reducing tinnitus symptoms, especially when personalized treatment plans are used. Despite positive outcomes in clinical trials, further research is needed to optimize stimulation parameters, understand the mechanisms at play, and refine therapeutic strategies for long-term relief. By addressing these gaps, bimodal neuromodulation has the potential to revolutionize tinnitus management, improving patient outcomes and quality of life for millions affected by this condition. Bimodal neuromodulation holds promise as a viable treatment for tinnitus and may also have broader applications in managing other chronic conditions.

## Data Availability

Data availability is not applicable to this article as no new data were created or analyzed in this study.
